# Physical fitness and enjoyment of physical activity among university students: implications for sustainable physical activity participation

**DOI:** 10.3389/fpsyg.2026.1772490

**Published:** 2026-03-20

**Authors:** Nan Zhang, Zhenzhen Su

**Affiliations:** 1 Physical Education College, Xi'an University of Architecture and Technology, Xi'an, Shaanxi, China; 2 School of Physical Education and Training, Xi’an Physical Education University, Xi’an, China

**Keywords:** higher education, physical activity enjoyment, physical education, physical fitness, sustainable physical activity participation, university students

## Abstract

**Background:**

Promoting sustainable physical activity participation among university students is a key challenge for higher education and public health systems. Enjoyment has been widely recognized as a critical psychological determinant of sustained engagement in physical activity; however, the role of physical fitness as an individual factor shaping exercise enjoyment remains insufficiently explored.

**Methods:**

This study examined differences in physical activity enjoyment between university students with high and low physical fitness levels. Undergraduate students (*N* = 80) were selected using an extreme-group design based on the National Student Physical Fitness Standards assessment. The top 15% (high-fitness group, *n* = 40) and bottom 15% (low-fitness group, *n* = 40) were matched for age and gender. Physical activity enjoyment was measured using the Physical Activity Enjoyment Scale (PACES). Independent-samples *t*-tests and effect size analyses were conducted.

**Results:**

The high-fitness group reported significantly higher enjoyment of physical activity (*M* = 103.45, SD = 17.80) than the low-fitness group (*M* = 77.68, SD = 19.31; *p* < 0.001), with a large effect size (Cohen’s d = 1.39). These findings indicate substantial differences in affective exercise experiences across fitness levels.

**Conclusion:**

Physical fitness is closely associated with enjoyment of physical activity among university students. Enhancing physical fitness and designing fitness-adaptive physical education programs may play a crucial role in fostering enjoyment and supporting sustainable physical activity participation in higher education.

## 1 Introduction

### 1.1 Physical fitness among university students

As China’s higher education system advances toward high-quality development, the physical and mental health of university students has become a fundamental component of talent cultivation and lifelong development (Ganley et al., 2011; Chen C. et al., 2020; Chen X. et al., 2020). Physical activity plays a central role in enhancing physiological function, alleviating psychological stress, and fostering health literacy (Rahmani-Nia et al., 2011; Sjostrom et al., 2006; Dong et al., 2023). Consequently, promoting sustainable participation in physical activity among university students has emerged as an important educational and public health priority.

Large-scale epidemiological evidence indicates a persistent decline in physical fitness among university students in China and worldwide (Cai et al., 2025; Huang et al., 2025; García-Lorenzo et al., 2024). Although overall fitness levels have decreased, substantial individual variation remains (Khandelwal and Gupta, 2014; Dregney et al., 2025), influenced by factors such as gender, socioeconomic status, and lifestyle (Kljajević et al., 2022). These disparities highlight the need to consider individual differences when designing strategies to promote long-term physical activity participation.

### 1.2 Physical activity enjoyment as a determinant of sustainable participation

Growing attention has been directed toward motivational processes underlying students’ engagement in physical activity (Biddle and Asare, 2011; Schneider et al., 2009; Teixeira et al., 2021). Among these mechanisms, enjoyment has consistently emerged as a key determinant of sustained exercise behavior. Physical activity enjoyment refers to positive affective responses associated with exercise participation, including pleasure, satisfaction, and interest (Wankel and Kreisel, 1985; Scanlan and Simons, 1992).

According to self-determination theory, enjoyment represents a core component of intrinsic motivation, which supports voluntary and persistent engagement in physical activity (Flannery, 2017; Teixeira et al., 2022). Individuals who experience enjoyment during exercise are more likely to incorporate physical activity into their routines and maintain participation over time, thereby contributing to behavioral sustainability (Yan et al., 2023; Chai et al., 2021).

### 1.3 Physical fitness and exercise-related experience

Although physical activity enjoyment has been widely studied, prior research has primarily focused on contextual influences such as activity type, instructional strategies, and social environments (Yan et al., 2023; Teixeira et al., 2022). Comparatively less attention has been devoted to physical fitness as an individual characteristic that may shape affective exercise experiences.

Theoretically, individuals with higher levels of physical fitness may perceive exercise as less physically demanding and more competence-enhancing, which may foster positive affect and enjoyment. Conversely, individuals with lower fitness levels may experience greater fatigue, discomfort, or perceived failure during exercise, potentially undermining positive emotional responses (Zheng et al., 2023; Borrego-Balsalobre et al., 2023). Empirical findings in this area remain limited and somewhat mixed, and the directional relationship between fitness and enjoyment has yet to be clarified, particularly in university populations. Recent competence-based developmental models suggest that lower physical fitness may initiate a self-reinforcing cycle of reduced perceived competence and weakened motivation, thereby shaping affective exercise experiences such as enjoyment (Zhang et al., 2026).

### 1.4 Research gap and study aim

Despite growing scholarly interest in both physical fitness and physical activity enjoyment, their direct interrelationship remains insufficiently examined. Most existing studies have investigated enjoyment as a predictor of physical activity behavior or fitness outcomes. In contrast, relatively few studies have directly compared levels of enjoyment across objectively differentiated fitness groups within a university context.

To address this gap, the present study compares physical activity enjoyment between university students with high and low physical fitness, as defined by standardized national fitness assessments. By integrating objective indicators of physical fitness with subjective measures of enjoyment, this study seeks to clarify whether meaningful affective differences are observable across distinctly different fitness profiles. In doing so, it aims to contribute evidence to discussions on enjoyment-driven, sustainable physical activity participation in higher education.

To facilitate a clear contrast between markedly different fitness levels, an extreme-group design was adopted. This approach is particularly appropriate when examining whether meaningful differences emerge at the extremes of a continuous individual characteristic. By maximizing between-group differentiation, the design enhances statistical sensitivity and allows clearer interpretation of potential affective differences. However, extreme-group comparisons may amplify observed effect sizes and limit generalizability to the broader student population. Accordingly, the findings should be interpreted with appropriate caution.

## 2 Materials and methods

### 2.1 Participants

A purposive sampling strategy was employed, with undergraduate students from Xi’an University of Architecture and Technology serving as the sampling frame. Participant screening was based on the National Student Physical Fitness Standards assessment conducted in April 2025. Ten classes ( N=298 ) were randomly selected, and students were ranked according to their overall physical fitness scores. To ensure a clear distinction in fitness levels, students in the top 15% were classified as the high-fitness group, while those in the bottom 15% were classified as the low-fitness group.

Based on the fitness score rankings of 298 students, approximately 45 students per group were initially identified in each extreme group (top 15% and bottom 15%). Some candidates were excluded due to incomplete physical fitness records or absence during questionnaire administration. In addition, to ensure balanced gender distribution between groups (male-to-female ratio = 1:1), further participants were excluded during the matching process. The final sample therefore consisted of 40 participants in the high-fitness group and 40 in the low-fitness group (total *N* = 80).

Eligibility criteria included: (a) complete physical fitness assessment data, (b) absence of major illness or injury, and (c) voluntary participation with written informed consent. The two groups were matched on key demographic variables. The mean age of the high-fitness group was 20.3 years ( SD=1.2) , while that of the low-fitness group was 20.5 years ( SD=1.1 ). Both groups had an equal gender distribution (male-to-female ratio = 1:1), thereby minimizing potential confounding effects of age and gender.

### 2.2 Physical fitness assessment

Physical fitness was assessed using the National Student Physical Fitness Standards (Ministry of Education of the People’s Republic of China, 2014), a nationally standardized evaluation system widely implemented across Chinese universities. This assessment framework has been extensively used in large-scale epidemiological monitoring and educational policy evaluation in China, ensuring consistency and comparability across institutions. The system comprises multiple dimensions reflecting body composition, physiological function, and physical performance.

The following test items were included: Body Composition (BMI). Height and weight were measured using standardized equipment, and body mass index (BMI) was calculated to assess body composition status. Physiological Function (Vital Capacity). Vital capacity was measured using a standardized spirometer in accordance with the National Student Physical Fitness Standards. Cardiorespiratory Endurance. Aerobic capacity was assessed using a 1,000-m run for males and an 800-m run for females, conducted on the university track. Muscular Strength. Lower-limb explosive strength was evaluated using the standing long jump test. Muscular Endurance. Muscular endurance was assessed using pull-ups for males and a 1-min sit-up test for females, representing upper-body and abdominal endurance, respectively. Flexibility. Flexibility of the lower back and hamstrings was assessed using the sit-and-reach test. Speed. Running speed was assessed using a 50-m sprint test, which evaluated short-distance acceleration and movement speed. All tests were administered in accordance with standardized national procedures, and the results were automatically recorded and archived in the university’s official health database.

Calculation of Overall Physical Fitness Score. The overall physical fitness score consisted of a weighted standard score (maximum = 100) plus potential bonus points (maximum = 20), yielding a total possible score of 120. The weighted standard score was calculated as follows: Total Score = (BMI × 15%) + (Cardiorespiratory Endurance × 20%) + (Muscular Strength × 10%) + (Muscular Endurance×10%) + (Flexibility × 10%) + (Speed × 10%). Bonus points were awarded for exceptional performance in selected indicators (pull-ups and the 1,000-m run for males; sit-ups and the 800-m run for females), with each indicator contributing up to 10 additional points. Based on the annual total score, students were classified into four levels: excellent (≥90.0), good (80.0–89.9), pass (60.0–79.9), and fail (<60.0).

All test data were obtained from the official database of the Health and Physical Fitness Center at Xi’an University of Architecture and Technology. Data integrity and compliance were verified by the university’s sports department prior to analysis.

### 2.3 Physical activity enjoyment scale

In the quantification of physical activity enjoyment, the Physical Activity Enjoyment Scale (PACES) stands as one of the most widely used and psychometrically sound assessment tools internationally. Initially developed by Kendzierski and DeCarlo (1991) for college student populations, this scale exhibits robust psychometric properties. The original study reported an exceptionally high internal consistency coefficient (Cronbach’s α = 0.96), demonstrating strong internal reliability; factor analysis further supported its structural validity. Additionally, PACES scores showed a significant positive correlation with relevant variables such as exercise motivation and self-efficacy, reflecting favorable discriminant and convergent validity—thus verifying its construct validity (Kendzierski and DeCarlo, 1991).

In recent years, the PACES has been extensively applied across diverse age groups and exercise contexts, including adolescents, adults, older adults, and various activity settings such as competitive sports, fitness training, and recreational exercise. Studies consistently confirm its stable reliability, validity, and good cross-cultural applicability (Moore et al., 2009; Motl et al., 2001; Mullen et al., 2011). Within Chinese research contexts, scholars have conducted preliminary localization, translation, and adaptation of the PACES, primarily for children and adolescent populations, validating its applicability and measurement quality in these groups (Sun, 2015; Guo et al., 2010; Chen et al., 2019). However, systematic validation specifically targeting Chinese college students remains scarce, and further examination is needed regarding its measurement structure, semantic comprehension, and psychometric properties within this distinct population.

To enhance the scientific rigor and adaptability of the measurement tool in the present study, we conducted a standardized translation of the English version of the PACES while preserving its original structure. We then administered acceptability testing and preliminary reliability and validity analyses in a small sample of college students. The scale comprises a set of statements describing individuals’ feelings during physical activity, such as “I find physical activity enjoyable,” “Exercise makes me feel pleasant,” and “I gain a sense of accomplishment from physical activity.” Participants rated each statement on a 7-point Likert scale based on their actual experiences (1 = Strongly Disagree, 7 = Strongly Agree), with total scores ranging from 18 to 126. Some items are reverse-coded (e.g., “I do not like physical activity”) and were recoded prior to scoring. The total score is calculated by summing the scores of all items, with higher total scores indicating greater enjoyment of physical activity. In the present sample ( N=80 ), the Chinese version of the PACES demonstrated excellent internal consistency (Cronbach’s α = 0.93).

### 2.4 Data collection procedure

Data collection was conducted in three stages: acquisition of physical fitness data, validation of the localized PACES scale, and administration of questionnaires. The detailed procedure is described below.

#### 2.4.1 Acquisition of physical fitness data

The researchers submitted a formal written application to the Director of the Health and Fitness Data Center at Xi’an University of Architecture and Technology. Upon approval, raw data from the National Student Physical Fitness Standards assessment for 10 target classes (*N* = 298) in 2025 were obtained. These data included individual test scores and overall fitness scores, which were used for participant grouping and fitness-level analyses.

#### 2.4.2 Validation of the localized PACES

The Physical Activity Enjoyment Scale (PACES; Kendzierski and DeCarlo, 1991) was first translated into Chinese by an experienced English instructor. Twenty bilingual undergraduate students were then recruited for validity testing. In Week 1, participants completed the English version of PACES; 1 week later, they completed the Chinese version. Score consistency between the two versions was examined using Pearson product–moment correlations. Results indicated correlation coefficients ranging from 0.76 to 0.91 (all *r* ≥ 0.70), meeting the psychometric criteria suggested by Nunnally and Bernstein (1994). These findings supported the validity of the Chinese version of PACES for use in the present study.

#### 2.4.3 Questionnaire administration

Potential participants were invited via email, which included a demographic survey. All students signed informed consent forms prior to participation. Questionnaires were administered immediately after a routine physical education class (50 min of moderate-intensity exercise) at the university stadium stands, ensuring that participants’ experiences of physical activity enjoyment remained salient. Research assistants provided standardized instructions, emphasizing that there were no right or wrong answers and encouraging honest responses. Completed questionnaires were collected anonymously and checked on site for completeness; careless or substantially incomplete responses were excluded.

Based on the overall physical fitness score rankings, valid samples were finalized, resulting in 40 participants in the high-fitness group and 40 in the low-fitness group. On average, participants required approximately 5 min to complete the questionnaire.

Throughout the data collection process, ethical principles of voluntary participation and confidentiality were strictly observed.

### 2.5 Data analysis

Data analysis was conducted using IBM SPSS Statistics 26.0. Means and standard deviations were calculated for the overall physical fitness scores and PACES scores of the high- and low-fitness groups to describe the distribution characteristics and confirm the effectiveness of the grouping procedure.

Independent-samples *t*-tests were conducted to compare PACES scores between the high- and low-fitness groups. Statistical significance was set at *p* < 0.05. Effect sizes were calculated using Cohen’s d to evaluate the magnitude of group differences.

### 2.6 Ethical considerations

This study involved human participants and was conducted in accordance with the Declaration of Helsinki and relevant institutional regulations. The research protocol was reviewed by the School of Physical Education at Xi’an University of Architecture and Technology and was determined to involve minimal risk and non-invasive procedures. Therefore, formal ethical approval was not required according to institutional guidelines. All participants were fully informed about the purpose and procedures of the study and provided written informed consent prior to participation. Participation was voluntary, and participants were free to withdraw at any time without penalty. All data were collected anonymously and treated confidentially.

## 3 Results

### 3.1 Descriptive statistics

Descriptive statistics for the individual physical fitness components are presented in Table 1. The high-fitness group demonstrated consistently better performance than the low-fitness group across all fitness indicators, including body composition, physiological function, cardiorespiratory endurance, muscular endurance, flexibility, and speed. These results confirm clear differentiation between the high- and low-fitness groups based on the composite physical fitness scores.

**Table 1:** Physical fitness component comparisons between high- and low-fitness groups.

Variable	High-fitness group (*n* = 40) Mean	SD	Low-fitness group (*n* = 40) Mean	SD
BMI (kg/m^2^)	20.60	2.14	29.47	5.60
Vital capacity (mL)	4,511	742	4,100	1,034
Cardiorespiratory endurance (1,000 m males/800 m females, s)	228.0	22.3	317.8	40.7
Standing long jump (cm)	242.8	30.5	180.5	29.0
Muscular endurance (pull-ups males/sit-ups females, reps)	18.9	9.3	3.9	9.2
Sit-and-reach (cm)	22.8	7.2	18.2	9.5
50 m sprint (s)	7.00	0.41	8.83	1.07

Values represent group means and standard deviations. Sex-specific tests were conducted according to the National Student Physical Fitness Standards (1,000 m run and pull-ups for males; 800 m run and sit-ups for females). The two groups were matched by gender (male-to-female ratio = 1:1).

**Figure 1:**
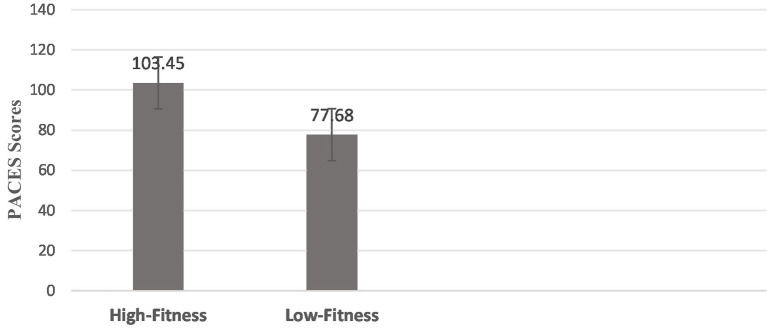
Comparison of PACES scores between high- and low-fitness groups. Bars represent mean PACES scores. Error bars represent ±1 standard deviation. ***indicates *p* < 0.001.

Descriptive statistics for the Physical Activity Enjoyment Scale (PACES) scores are presented in Table 2. The high-fitness group had a mean PACES score of 103.45 ( SD=17.80) , whereas the low-fitness group had a mean score of 77.68 ( SD=19.31) . The mean difference between groups was 25.77 points. The distribution of mean PACES scores by fitness group is visually presented in Figure 1.

**Table 2:** Descriptive statistics and independent-samples *t* test results for PACES scores by fitness group.

Group	*n*	*M*	SD	*t*	df	*p*	95% CI of mean difference	Cohen’sd
High-fitness group	40	103.45	17.80	6.22	78	<0.001	[17.54, 34.06]	1.39
Low-fitness group	40	77.68	19.31					

M, mean; SD, standard deviation; CI, confidence interval.

The standard deviation in the high-fitness group ( SD=17.80 ) was slightly smaller than that of the low-fitness group ( SD=19.31 ). To further confirm group differentiation, overall physical fitness scores were also examined. The high-fitness group had a mean fitness score of 89.95 ( SD=5.61 ), whereas the low-fitness group had a mean score of 61.77 ( SD=6.72 ).

### 3.2 Independent *t*-test results

An independent-samples *t*-test was conducted to examine differences in PACES scores between the high-fitness and low-fitness groups. Levene’s test indicated that the assumption of homogeneity of variances was not violated ( F=0.096,p=0.758 ). The *t*-test revealed a statistically significant difference in PACES scores between the two groups, *t*(78) = 6.22, *p* < 0.001 (two-tailed). The 95% confidence interval for the mean difference ranged from 17.54 to 34.06.

### 3.3 Effect size analysis

Cohen’s d was calculated to estimate the standardized mean difference between the two groups (Cohen, 1988). The effect size was *d* = 1.39. The 95% confidence interval for the effect size ranged from 0.90 to 1.88.

## 4 Discussion

### 4.1 Summary of findings and theoretical interpretation

The present study examined differences in physical activity enjoyment between university students at opposite ends of the physical fitness spectrum. The results indicate that students with higher levels of physical fitness reported significantly greater enjoyment during physical activity than their lower-fitness counterparts. These findings provide empirical evidence that physical fitness is meaningfully associated with affective exercise experiences in a university population.

From a theoretical perspective, this association may be understood through the lens of intrinsic motivation and perceived competence. According to self-determination theory (Deci and Ryan, 1985), enjoyment represents a central component of intrinsic motivation and plays a pivotal role in sustaining voluntary engagement in physical activity. When exercise experiences are accompanied by pleasure and satisfaction, individuals are more likely to maintain participation over time (Flannery, 2017; Andrews, 1903; Henderson et al., 2004).

Students with higher physical fitness typically possess greater motor proficiency and physiological capacity, enabling them to perform exercise tasks with reduced strain and greater efficiency. Such advantages may strengthen perceptions of competence and mastery, which are closely linked to positive affective responses. In contrast, students with lower fitness levels may encounter greater physical difficulty during exercise, potentially diminishing enjoyment and contributing to negative emotional experiences. This interpretation aligns with previous evidence suggesting that positive affect declines when exercise demands exceed individual capacity (Teixeira et al., 2022).

Overall, the findings underscore the relevance of physical fitness as an individual-level factor associated with exercise enjoyment. Rather than viewing fitness solely as a physical outcome, the present results highlight its potential psychological correlates and suggest that fitness level may shape the qualitative experience of physical activity, which is central to sustainable participation in university settings.

### 4.2 Comparison with previous research

The findings of the present study are broadly consistent with prior research while extending the literature through a direct extreme-group comparison of objectively assessed fitness levels and subjective enjoyment within a university context. Previous studies have primarily examined enjoyment as a predictor of physical activity behavior or fitness outcomes (Calder et al., 2020; Ohashi et al., 2023; Hagberg et al., 2009; Henderson et al., 2004; Costigan et al., 2024). In contrast, the present study reverses this perspective by examining whether individuals at distinctly different fitness levels report differential enjoyment, thereby contributing a complementary viewpoint to the existing body of research.

The observed effect size (Cohen’s d = 1.39) reflects a substantial standardized difference between groups, indicating a marked separation in reported enjoyment across contrasting fitness profiles within the sampled population. Nonetheless, this magnitude should be interpreted with consideration of the extreme-group design, which maximizes between-group contrast and may yield larger standardized differences than would be observed in a full-range sample.

Furthermore, the present results align with findings from adolescent populations, in which positive associations between physical fitness and subjective exercise experiences have been documented (Motl et al., 2001; Ha et al., 2024; Borrego-Balsalobre et al., 2023). By focusing on university students, this study extends prior evidence to a later developmental stage, suggesting that the association between fitness and enjoyment may persist beyond adolescence.

At the same time, the relationship between physical fitness and enjoyment is likely dynamic and potentially bidirectional. Higher fitness may enhance enjoyment through increased perceived competence and reduced physiological strain, whereas enjoyment may motivate sustained engagement, thereby contributing to subsequent improvements in fitness. This reciprocal dynamic—conceptually grounded in motivational frameworks such as self-determination theory—has been proposed in longitudinal and intervention research. Although the present cross-sectional design precludes causal inference, the findings are consistent with this mutually reinforcing conceptual framework.

### 4.3 Limitations and challenges

Several limitations of this study should be acknowledged. First, the sample was drawn from a single university, and each group included a relatively small number of participants. Although the extreme-group design enhanced between-group differences, it limits the generalizability of the findings to the broader population of university students. Second, the cross-sectional nature of the study precludes causal inference. It remains unclear whether higher physical fitness leads to greater enjoyment or whether students who enjoy physical activity are more likely to develop higher fitness levels. Longitudinal and experimental designs are needed to clarify this relationship. Third, although physical fitness was assessed using standardized objective measures, enjoyment was measured through self-report, which may be subject to response bias. In addition, standardized fitness assessments may not fully capture broader aspects of physical health, such as psychological resilience or lifestyle behaviors.

### 4.4 Future research directions

Future research should adopt longitudinal and intervention-based designs to examine the dynamic relationship between physical fitness and exercise enjoyment. For example, enjoyment-oriented interventions—such as gamified activities or low-threshold recreational programs—could be implemented among low-fitness students to assess whether enhanced enjoyment leads to improved participation and fitness outcomes. Conversely, fitness-enhancement programs could examine whether improvements in physical capacity subsequently increase enjoyment.

Such approaches would provide a more comprehensive understanding of how enjoyment and fitness interact over time and contribute to sustainable physical activity participation. These efforts align with the broader goals of sustainable health promotion and may inform strategies to support both physical and psychological well-being among university students.

## 5 Conclusions and recommendations

### 5.1 Conclusion

This study identified significant differences in physical activity enjoyment between university students with high and low physical fitness levels. Students with higher fitness reported substantially greater enjoyment, with a large effect size, indicating a meaningful association between physical fitness and affective exercise experience.

The findings suggest that physical fitness may be linked to differential exercise experiences, potentially shaping patterns of motivation and participation. Students with higher fitness levels may be more likely to experience positive reinforcement through competence and enjoyment, whereas those with lower fitness levels may face barriers related to reduced enjoyment. Although causal direction cannot be established, the results are consistent with theoretical models emphasizing the reciprocal relationship between enjoyment and physical activity behavior.

### 5.2 Implications for sustainable physical education

The observed differences in enjoyment across fitness levels highlight a central challenge for physical education: balancing standardized curriculum requirements with individual differences. Programs that prioritize uniform performance benchmarks may unintentionally disadvantage students with lower fitness while insufficiently engaging those with higher fitness.

From a sustainability perspective, physical education in higher education should prioritize long-term engagement over short-term performance outcomes (Teixeira et al., 2022). This requires instructional approaches that support positive exercise experiences across diverse fitness profiles (Khandelwal and Gupta, 2014; Ohashi et al., 2023; Williams, 2013).

#### 5.2.1 Reframing the value orientation of physical education

Physical education should not equate physical fitness solely with athletic performance (Wang, 2020). Enjoyment is shaped not only by task completion but also by perceived competence, safety, and personal achievement. Students with lower fitness levels may benefit from adapted activities that promote attainable success experiences, while students with higher fitness levels require appropriate challenges to sustain engagement. Recognizing fitness-related differences as variations in experience rather than deficits is essential for inclusive participation.

#### 5.2.2 Designing fitness-adaptive instruction

Fitness-adaptive strategies may be implemented across curriculum design, activity selection, evaluation systems, and classroom climate. Tiered instructional models that pursue shared objectives through differentiated pathways can better accommodate diverse fitness levels. Expanding activity options beyond traditional competitive sports may enhance inclusivity. Evaluation systems that emphasize individual progress and participation, rather than rigid benchmarks, can further support sustained engagement. Additionally, supportive classroom environments that foster peer encouragement and minimize social comparison may reduce anxiety and enhance enjoyment.

#### 5.2.3 Toward sustainable participation

Ultimately, differences in exercise enjoyment should be viewed as signals for instructional adaptation rather than student limitation. The primary mission of physical education in higher education is not elite performance but the cultivation of confidence, enjoyment, and sustained participation in physical activity. By creating environments that enable all students to experience competence and engagement, universities can contribute meaningfully to long-term health and well-being.

## Data Availability

The original contributions presented in the study are included in the article/supplementary material, further inquiries can be directed to the corresponding author.
